# Dietary Macroalgae *Saccharina japonica* Ameliorates Liver Injury Induced by a High-Carbohydrate Diet in Swamp Eel (*Monopterus albus*)

**DOI:** 10.3389/fvets.2022.869369

**Published:** 2022-06-14

**Authors:** Chuanqi Yu, Lu Wang, Wanghe Cai, Wenping Zhang, Zhonghua Hu, Zirui Wang, Zhuqing Yang, Mo Peng, Huanhuan Huo, Yazhou Zhang, Qiubai Zhou

**Affiliations:** ^1^College of Animal Science and Technology, Jiangxi Agricultural University, Nanchang, China; ^2^Key Laboratory of Featured Hydrobios Nutritional Physiology and Healthy Breeding, Nanchang, China

**Keywords:** dietary macroalgae, high-carbohydrate diet, fresh-water fish, liver injury, multiple omics

## Abstract

A high-carbohydrate diet lowers the rearing cost and decreases the ammonia emission into the environment, whereas it can induce liver injury, which can reduce harvest yields and generate economic losses in reared fish species. Macroalgae *Saccharina japonica* (SJ) has been reported to improve anti-diabetic, but the protective mechanism of dietary SJ against liver injury in fish fed a high-carbohydrate diet has not been studied. Therefore, a 56-day nutritional trial was designed for swamp eel *Monopterus albus*, which was fed with the normal diet [20% carbohydrate, normal carbohydrate (NC)], a high carbohydrate diet (32% carbohydrate, HC), and a HC diet supplemented with 2.5% SJ (HC-S). The HC diet promoted growth and lowered feed coefficient (FC), whereas it increased hepatosomatic index (HSI) when compared with the NC diet in this study. However, SJ supplementation increased iodine contents in muscle, reduced HSI, and improved liver injury, such as the decrease of glucose (GLU), total bile acid (TBA), and alanine aminotransferase (ALT) in serum, and glycogen and TBA in the liver. Consistently, histological analysis showed that SJ reduced the area of lipid droplet, glycogen, and collagen fiber in the liver (*p* < 0.05). Thoroughly, the underlying protective mechanisms of SJ supplementation against HC-induced liver injury were studied by liver transcriptome sequencing coupled with pathway analysis. The Kyoto Encyclopedia of Genes and Genomes (KEGG) pathway enrichment analysis of the differentially expressed genes (DEGs), such as the acetyl-coenzyme A synthetase *(acss1)*, alcohol dehydrogenase (*adh)*, interferon-induced protein with tetratricopeptide repeats 1 (*ifit1)*, aldo-keto reductase family 1 member D1 (*akr1d1*), cholesterol 7-alpha-monooxygenase *(cyp7a1)*, and UDP-glucuronosyltransferase *(ugt)*, indicated that the pathway of glycolysis/gluconeogenesis was the main metabolic pathway altered in the HC group compared with the NC group. Meanwhile, hepatitis C, primary BA biosynthesis, and drug metabolism-cytochrome P450 were the three main metabolic pathways altered by SJ supplementation when compared with the HC group. Moreover, the BA-targeted metabolomic analysis of the serum BA found that SJ supplementation decreased the contents of taurohyocholic acid (THCA), taurochenodeoxycholic acid (TCDCA), taurolithocholic acid (TLCA), nordeoxycholic acid (NorDCA), and increased the contents of ursocholic acid (UCA), allocholic acid (ACA), and chenodeoxycholic acid (CDCA). In particular, the higher contents of UCA, ACA, and CDCA regulated by SJ were associated with lower liver injury. Overall, these results indicate that the 2.5% supplementation of SJ can be recommended as a functional feed additive for the alleviation of liver injury in swamp eel-fed high-carbohydrate diets.

## Introduction

Carbohydrates are regarded as the most economical energy source for aquatic animals due to their abundance and relatively low cost (1). However, it is generally acknowledged that fish have a poor capability to utilize glucose (GLU) for energy purposes compared with mammals (2). Generally, most fish species (especially carnivorous ones) have an impaired GLU tolerance and often display prolonged postprandial hyperglycemia after a GLU load or the intake of a high-carbohydrate diet (3). It has additionally been reported that a high-carbohydrate diet induces liver injury, which can result in severe health problems and reduce harvest yields, thus generating economic losses in reared fish (1, 4). Therefore, the amelioration of liver injury is strongly necessary for the healthy development of aquaculture.

*Saccharina japonica* (SJ), a common macroalgae cultured in the temperate coastal area of the northwest Pacific Ocean, is widely cultured and has become the most productive seaweed in China (5). In particular, SJ has been reported to exhibit various biological activities, such as improving immunity, anti-tumor, and anti-diabetic, which are ascribed to the variety of biologically active ingredients, such as polysaccharides, polyphenols, vitamins, and minerals (6, 7). In recent years, dietary SJ has been shown to ameliorate liver injury by decreasing hepatic collagen fiber and serum aspartate aminotransferase (AST) and alanine aminotransferase (ALT) in mammals (7, 8). Moreover, studies have demonstrated that liver injury is commonly characterized by increased levels of total bile acid (TBA) and triglyceride (TG), AST, and ALT in the plasma (8–10). However, information regarding the effects of dietary SJ on liver injury in the fish fed high-carbohydrate diet is not reported.

Swamp eel (*Monopterus albus*) is a high-value commercially farmed, eurythermal, and freshwater carnivorous fish with a desirable growth rate, good meat quality, and an exceptional ability to obtain oxygen from the air rather than water (11, 12). Our previous study showed that a high-carbohydrate diet resulted in liver injury in swamp eel (13).

This study aimed to investigate the effect of dietary SJ on high-carbohydrate diet-induced liver injury in swamp eel by analyzing serum and hepatic biochemistry, and morphology of the liver in the present study. The liver genes and serum bile acid profile were also studied by the transcriptomic and metabolomic analysis.

## Materials and Methods

### Experimental Diets and Feeding Trial

Dry macroalgae SJ meal was purchased from Qingdao Longan Biotechnology Co., Ltd (Qingdao, China). Three isonitrogenous and isolipidic diets were formulated: the normal carbohydrate diet (20% carbohydrate, NC), high carbohydrate diet (32% carbohydrate, HC), and HC diet supplemented with 2.5% SJ (HC-S) (Table 1). The NC level of 20% and HC level of 32% were obtained in our previous study (unpublished data). This SJ supplementation level was selected based on our previous study (14) and relevant publications in the field (7). The diet was prepared, and ingredients were ground, weighed, and well-mixed as previously described (15).

**Table 1:** Formulation and proximate composition of the experimental diet used in this feeding trial.

**Ingredients (g/100 g)**	**NC**	**HC**	**HC-S**
Fishmeal	40.00	40.00	40.00
Shrimp meal	2.00	2.00	2.00
Earthworm meal	2.00	2.00	2.00
Spray-dried blood meal	5.00	4.25	4.25
Soy protein concentrate	12.75	12.75	12.75
low-gluten flour	20.00	32.00	32.00
Microcrystalline cellulose	13.75	2.50	0.00
^b^*Saccharina japonica* meal			2.50
Monocalcium phosphate	1.00	1.00	1.00
^a^Mineral and vitamin premix	1.00	1.00	1.00
Phytase	0.25	0.25	0.25
Soybean oil	2.25	2.25	2.25
**Proximate composition, % dry matter**			
Moisture	10.32	10.61	10.54
Crude protein	43.06	43.58	43.79
Crude lipid	7.15	7.54	7.54
Ash	10.74	10.98	11.38

*^a^Vitamins (mg or IU/kg diet): vitamin D_3_, 2,500.00 IU; vitamin E, 200.00 mg; vitamin K_3_, 10.00 mg; vitamin B_1_, 25.00 mg; vitamin B_2_, 45.00 mg; nicotinic acid, 200.00 mg; vitamin B_6_, 20.00 mg; Ca-pantothenate acid, 60.00 mg; folic acid, 10.00 mg; vitamin B_12_, 0.10 mg; biotin, 1.5 mg; vitamin C, 200.00 mg; inositol, 200.00 mg. ^a^Minerals (g or mg/kg diet): NaSeO_3_·5H_2_O, 0.30 mg; CoCl_2_·6H_2_O, 0.40 mg; KI, 0.80 mg; CuSO_4_·5H_2_O, 10.00 mg; MnSO_4_·4H_2_O, 20.0 mg; ZnSO_4_·H_2_O, 50.00 mg; FeSO_4_·7H_2_O, 150.00 mg; MgSO_4_·7H_2_O, 500.00 mg; NaCl, 1000.00 mg*.

*^b^Saccharina japonica (SJ) meal (g/100g): Crude protein, 8.20; Crude lipid, 0.30; total dietary fiber, 12.43; K, 8.68; Na, 3.46; Ca, 1.01; Mg, 0.32; P, 0.18; I, 4.01; Qingdao, China*.

The 56-day feeding trial were conducted according to our previous study with some modification (11). Each diet group was randomly assigned to four replicates, and one replicate corresponded to one tank (100 cm × 60 cm × 50 cm, 20 healthy swamp eels per tank with an initial weight of 12.10 ± 0.03 g). Fish were fed with the experimental diet to apparent satiation once daily (at 17:00) referring to our previous study (14) and the feed intake was recorded. During the experiment, the water temperature ranged from 27 to 32°C, ammonia nitrogen was lower than 3 mg/L, and dissolved oxygen was above 4 mg/L.

### Sample Collection

At the end of the feeding trial, all fish were counted and weighed after fasting for 12 h and anesthetized with 250 mg/L M-aminobenzoate ethyl methane sulfonate (MS-222) (Sigma-Aldrich, USA). A total of 16 fish (4 fish per tank) were sampled from four tanks in each group (*n* = 4). Blood was collected from the caudal vein using a 1-ml syringe. Afterward, the fish was dissected on ice to obtain dorsal muscle and liver subsequently. After dissection, the liver was cut into small pieces. After sedimentation at 4°C for 2 h, pooled blood samples were centrifuged at 3,500 g for 15 min at 4°C to obtain serum. Parts of the liver were fixed in 4% buffered neutral formalin for histological analysis. Dorsal muscle, the other parts of the liver and serum were immediately frozen in liquid nitrogen for 6 h and stored at −80°C for transcriptomic, metabolomics, and biochemical analysis. Samples were kept in 1.5 ml plastic tubes.

Survival rate (SR) = 100 × (final fish number/initial fish number). Weight gain rate (WGR) = 100 × (final body weight—initial body weight)/(initial body weight). Feed coefficient (FC) = (total dry weight of feed fed)/(final weight—initial weight). Hepatosomatic index (HSI) = 100 × liver weight/body weight.

### Proximate Composition and Iodine Analysis

Moisture, ash, crude protein, and lipid were tested following the methods of the Association of Official Analytical Chemists (AOAC 2,000) with the analytical numbers 950.46, 960.39, 928.08, and 920.153, respectively. Moisture was determined by drying ground samples in a forced-air oven at 105°C for 24 h. Ash was analyzed by incinerating samples at 600°C for 24 h in a muffle furnace. Crude protein was estimated as Kjeldahl-nitrogen using a factor of 6.25, and crude lipid was analyzed by Soxhlet extraction with petroleum ether. Iodine content was determined at Guangdong Kangxin Testing Technology Co., Ltd. (Guangzhou, China) by microwave digestion with nitric acid and hydrogen peroxide (Germany Merck superior grade pure), followed by inductively coupled plasma mass spectrometry (Agilent ICPMS-7900) detection.

### Biochemistry Analysis

The liver sample was accurately weighed, and 9 times the volume of physiological saline was added. Then, it was mechanically homogenized under ice-water bath conditions and centrifuged at 350 g for 10 min, and the supernatant was collected. Both the supernatants of the liver and serum were used for biochemistry analysis. AST and ALT activities, TG, TBA, GLU, insulin, and glycogen contents were determined using the diagnostic reagent kits for fish (Nanjing Jiancheng Bioengineering Institute, China) according to the manufacturer's instructions. In brief, a volumetric supernatant was transferred to a 96-well plate, then the reaction solution was added to each sample. The 96-well plate was incubated at specific conditions, and then the 96-well plate was measured with the microplate spectrophotometer (Spectra Max^®^ 190).

### Histological Analysis

The fixed hepatic tissues were processed with the standard paraffin embedding method and then stained with Oil Red O (making nucleus blue and lipid red), PAS (making glycogen carmine), and Masson (making collagen fiber blue), respectively. The slides were then examined and photographed under a light microscope (Axio Imager 2, Zeiss, Oberkochen, Germany) equipped with a camera (Axiocam 506, Zeiss, Oberkochen, Germany). In addition, the relative area of lipid droplet, glycogen, and collagen fiber in the liver from 16 fish per group was measured using Image-Pro Plus 6.0 (*n* = 16).

### Transcriptomic Assay

Total RNA was extracted from the liver tissues of the control group (NC), high carbohydrate group (HC), and HC diet supplemented with 2.5% SJ group (HC-S) using Trizol reagent (Invitrogen, USA) according to the manufacturer's instructions (12 fish per group). The Nanodrop 2,000 (Thermo Fiser Scientific, Wilmington, DE) was used to determine RNA purity and concentration. The integrity of the total RNA was assessed by the RNA Nano 6,000 assay kit of the Agilent 2,100 system (Agilent Technologies, CA, USA). Three Illumina libraries, each containing a pool of equal total RNA from four individual samples, were produced for each group (NC, HC, and HC-S). The messenger RNA (mRNA) was purified from the total RNA using poly-T oligo-attached magnetic beads, and then broken into short fragments with fragmentation buffer. First, the mRNA fragments were used as templates for the synthesis of complementary DNA (cDNA). Second, cDNA fragments of preferentially 240 bp in length, the library fragments were purified with the AMPure XP system (Beckman Coulter, Beverly, USA). At last, the suitable fragments were used for PCR amplification, and PCR products were purified (AMPure XP system). The library quality was assessed on the Agilent Bioanalyzer 2,100 system. The library was sequenced on an Illumina Hiseq X Ten platform (Illumina, CA, USA). The genome data from NCBI of swamp eel *Monopterus albus* were used for further analysis in this study.

Gene expression levels were estimated by fragments per kilobase of transcript per million fragments mapped (FPKM). Differentially expressed genes (DEGs) between groups were verified by DESeq2. According to fold change (FC) ≥1.5 and false discovery rate (FDR) value <0.05, DEG in the liver between different groups was identified. DEG was submitted to the KEGG database (http://www.genome.ad.jp/kegg/) to come up with pathways in KEGG pathway categories. The statistical enrichment of DEGs in the KEGG pathway was tested using KOBAS software.

### Gene Expression Validation

The quantitative real-time PCR (qPCR) was performed using SYBR Green as fluorescent dye according to the manufacturer's protocol (Takara), which was used to validate and quantify genes, such as *adh* (alcohol dehydrogenase), *cyp7a1* (cholesterol 7-alpha-monooxygenase), *akr1d1* (aldo-keto reductase family 1 member D1), *acss1* (acetyl-coenzyme A synthetase), *ifit1* (interferon-induced protein with tetratricopeptide repeats 1), and *ugt* (UDP-glucuronosyltransferase) from the transcriptomic assay. The primers used for qPCR are listed in Supplementary Table 1. Each sample was run in triplicate. The 2^−Δ*ΔCt*^ method was used to calculate the relative expression with β-actin as a reference gene.

### BA Composition by Ultra-Performance Liquid Chromatography-Mass Spectrometry Analysis

Blood samples of the NC, HC, and HC-S groups were put in the EP tube, and 600 μl methanol (−20°C) was added (16 fish per group). After the 60 s of the vortex, the samples were centrifuged at 12,000 rpm for 10 min at 4°C. A volume of 400 ul of supernatant was transferred to a fresh tube and dried in a vacuum. Then, the dried samples were dissolved with 100 μl of 30% methanol (−20°C). Finally, the supernatant was filtered through a 0.22 μm membrane for ultra-performance liquid chromatography-mass spectrometry (UPLC-MS) analysis.

In the UPLC-MS experiment, UPLC separation was performed on an Acquity UPLC system (Waters, U.K.) equipped with an Acquity UPLC^®^ BEH C18 (1.7 μm, 2.1 mm × 100 mm, Waters) column. The temperature of the column was set at 40°C. The sample injection volume was 5 μl. Eluents consisted of 0.01% formic acid water (eluent A) and acetonitrile (eluent B). The flow rate was set at 0.25 ml/min. A 35.5-min elution gradient was performed as follows: 0–4 min, 25% B; 4–9 min, 25–30% B; 9–14 min, 30–36% B; 14–18 min, 36–38% B; 18–24 min, 38–50% B; 24–32 min, 50–75% B; 32–33 min, 75–90% B; and 33–35.5 min, 90–25% B.

The MS analysis was performed by an AB mass spectrometer (AB, USA) equipped with an ESI source in the negative-ion mode working in the multiple reaction monitoring (MRM) mode. An ion source voltage of 4,500 V and a source temperature of 500°C were used.

The calibration graphs were constructed by plotting the peak area vs. concentration for each standard. The results were shown in the report of analysis and the correlation coefficients were >0.99. The limit of quantitation (LOQ) was determined by the signal-to-noise ratio (S/N), which was calculated by comparing the signals of known samples with blank samples. Generally, the corresponding concentration was defined as LOQ when the S/N is 10:1 (S/N = 10). The precision of the analytical procedure was expressed as the relative standard deviation (RSD). The intra- and interday precision was 1.52–10.14% and 2.18–23.44%, respectively. It indicated that the instrument is of good precision.

### Statistical Analysis

The data for each group were expressed as mean ± standard error of mean (SEM). SPSS 19.0 software was used for one-way ANOVA, and then Tukey's multi-range test was used to evaluate the statistical differences in growth, composition, histology, biochemistry analysis, and qPCR between treatments. The level of significance was set as *p* < 0.05.

## Results

### Growth Performance and Feed Utilization of Swamp eel Fed NC, HC, and HC-S Diet

Growth performance and feed utilization of swamp eel are presented in Table 2. The final body weight (FBI), survival rate (SR), and weight gain rate (WGR) showed non-significant differences between groups (*p* > 0.05). A significantly lower feed coefficient (FC) was found in the HC group compared with the NC group (*p* < 0.05). However, fish in HC and HC-S groups had significantly higher HSI than that of fish in the NC group (*p* < 0.05), and SJ supplementation inhibited the abnormal growth of the liver caused by the HC diet (*p* > 0.05).

**Table 2:** Effects of dietary SJ on growth performance and feed utilization swamp eel fed high-carbohydrate (HC) diet.

**Group**	**^**1**^IBW, g**	**^**2**^FBW, g**	**^**3**^SR, %**	**^**4**^WGR, %**	**^**5**^FC**	**^**6**^HSI**
NC	12.11 ± 0.09	40.21 ± 0.61	100 ± 0.00	231.99 ± 4.7	1.16 ± 0.02^*a*^	5.9 ± 0.37^*b*^
HC	12.10 ± 0.07	41.63 ± 0.82	100 ± 0.00	244.2 ± 8.59	1.04 ± 0.03^*b*^	7.54 ± 0.23^*a*^
HC-S	12.18 ± 0.05	39.49 ± 0.65	100 ± 0.00	224.28 ± 5.56	1.13 ± 0.02^*ab*^	6.95 ± 0.08^*a*^

*Data are presented as means ± SEM, n = 4 tanks (20 fish/tank)*.

*^a, b^Data with different superscript letters in the same line are significantly different (p < 0.05)*.

*^1^IBW, initial body weight*.

*^2^FBW, final body weight*.

*^3^SR, survival rate = 100 × (final fish number/initial fish number)*.

*^4^WGR, weight gain rate (%) = 100 × (final body weight—initial body weight)/(initial body weight)*.

*^5^FC, feed coefficient = (total dry weight of feed fed)/(final weight—initial weight)*.

*^6^HSI, hepatosomatic index = 100 × liver weight/body weight*.

### Muscle Nutritional Values, and Hepatic and Serum Biochemistry of Swamp eel Fed NC, HC, and HC-S Diet

There was no significant difference in moisture, crude protein, crude lipid, and ash content between groups (*p* > 0.05). However, significantly higher iodine content was found in the HC-S group compared with the NC and HC groups (*p* < 0.05) (Table 3). Tissue biochemical parameters of swamp eel are presented in Table 4. Serum TBA, GLU, TG, and AST, and hepatic TBA, TG, and glycogen levels in the HC group were significantly higher than those in the NC group (*p* > 0.05), whereas the HC-S diet lower or significantly lower than those in the swamp eel fed HC diet. However, serum insulin levels showed no significant difference between groups (*p* > 0.05).

**Table 3:** Effects of dietary SJ on muscle nutritional values of swamp eel fed HC diet.

**Parameter**	**NC**	**HC**	**HC-S**
Moisture (g/100 g)	74.69 ± 0.52	73.7 ± 0.28	73.81 ± 0.48
Protein (g/100 g)	19.42 ± 0.53	20.32 ± 0.2	21.03 ± 0.45
Lipid (g/100 g)	2.47 ± 0.08	2.8 ± 0.15	2.66 ± 0.08
Ash (g/100 g)	1.05 ± 0.08	1.07 ± 0.01	1.16 ± 0.01
Iodine (mg/1000 g)	0.64 ± 0.01^b^	0.86 ± 0.02^b^	22.21 ± 0.74^a^

*Data are presented as means ± SEM, n = 4 tanks (20 fish/tank)*.

*^a, b^Data with different superscript letters in the same line are significantly different (p < 0.05)*.

**Table 4:** Effects of dietary SJ on hepatic and serum biochemistry of swamp eel fed HC diet.

**Tissue**	**Parameter**	**NC**	**HC**	**HC-S**
Serum	Glucose (GLU), mmol/L	3.8 ± 0.10^c^	7.12 ± 0.18^a^	6.01 ± 0.17^b^
	Triglyceride (TG), mmol/L	2.21 ± 0.18^b^	2.78 ± 0.11^a^	2.31 ± 0.16^ab^
	Aspartate aminotransferase (AST), U/L	6.72 ± 0.38^b^	12.54 ± 0.71^a^	11.53 ± 0.41^a^
	Alanine aminotransferase (ALT), U/L	1.25 ± 0.02^a^	1.26 ± 0.02^a^	1.14 ± 0.01^b^
	Insulin, U/L	6.36 ± 0.38	6.38 ± 0.26	6.81 ± 0.25
	Total bile acid (TBA), umol/L	30.98 ± 1.07^b^	40.16 ± 1.85^a^	33.67 ± 0.68^ab^
Liver	Triglyceride (TG), x 10^−2^mmol/gprot	0.88 ± 0.04^c^	3.23 ± 0.08^a^	1.41 ± 0.09^b^
	Glycogen, mg/g	19.49 ± 1.13^b^	22.66 ± 0.41^a^	21.02 ± 0.53^ab^
	Total bile acid (TBA), umol/L	85.86 ± 1.47^c^	226.55 ± 6.20^a^	167.77 ± 5.41^b^

*Data are presented as means ± SEM, n = 4 tanks (20 fish/tank)*.

*^a−c^Data with different superscript letters in the same line are significantly different (p < 0.05)*.

### Histological Structure in Liver of Swamp eel Fed NC, HC, and HC-S Diet

**Figure 1:**
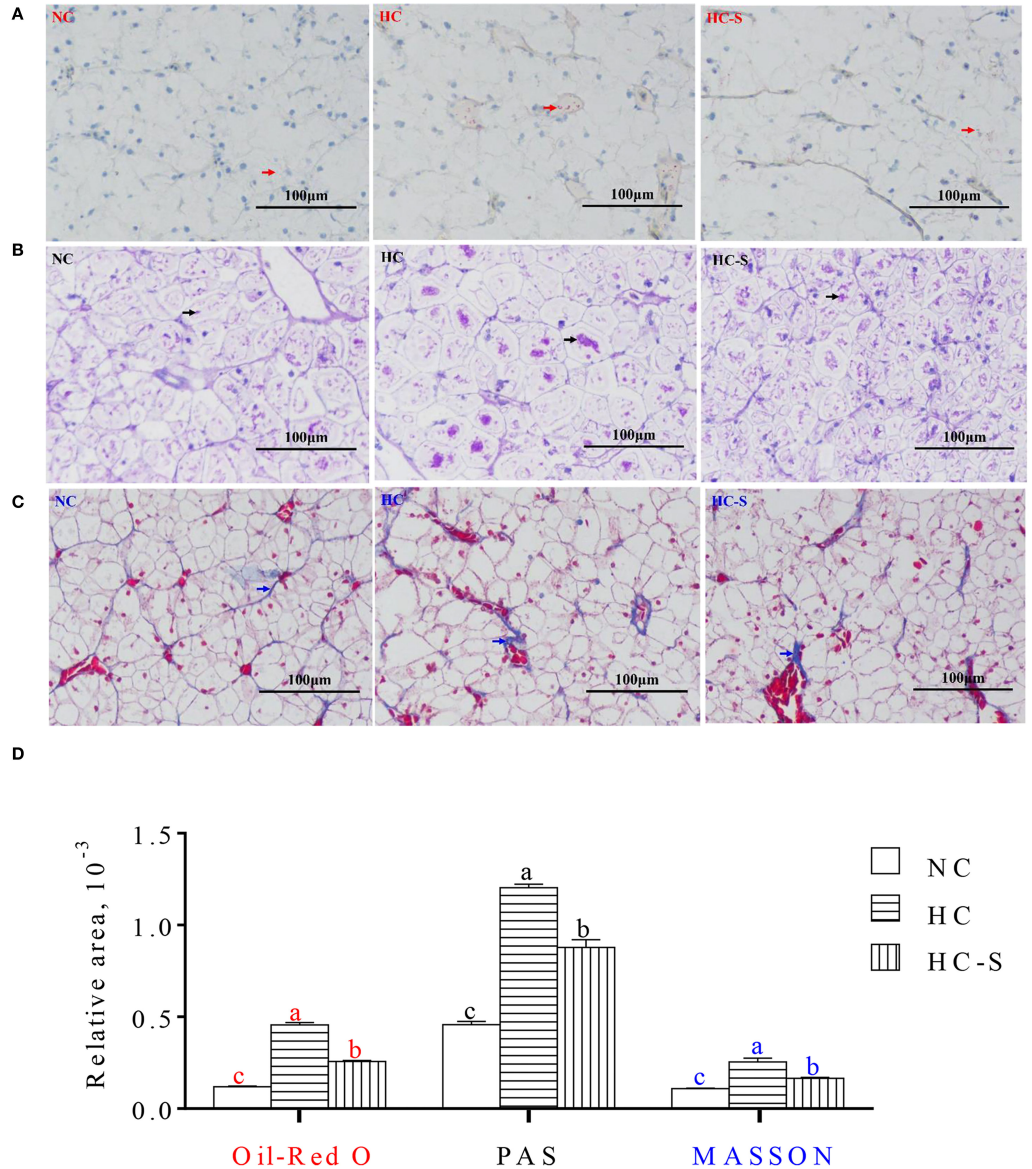
Influence of dietary *Saccharina japonica* (SJ) on hepatic histological structure of paraffin slice with Oil Red O (making nucleus blue and lipid red) staining **(A)**, PAS (making glycogen carmine) staining **(B)** and Masson (making collagen fiber blue) staining **(C)**, and the relative areas of lipid droplet with red arrows in Oil-Red O staining, glycogen with black arrows in PAS staining, and collagen fiber with blue arrows in MASSON staining of swamp eel fed high-carbohydrate (HC) diet **(D)** ( × 200). Values are presented as means ± SEM, *n* = 16 fish/diet. Means with different letters were significantly different (*p* < 0.05).

The relative areas of lipid droplet (in Oil-Red O stained), glycogen (in PAS stained), and collagen fiber (in MASSON stained) from fish liver paraffin slice were significantly increased in the HC group compared with the NC group, whereas the HC-S group significantly reduced the relative areas of lipid droplet, glycogen, and collagen fiber compared with the HC group (*p* < 0.05) (Figure 1).

### Liver Transcriptome Profile of Swamp eel Fed NC, HC, and HC-S Diet

RNA samples were extracted from the liver tissues of NC, HC, and HC-S groups for RNA sequencing, and generated 21.53, 21.39, and 20.88 million clean reads, respectively (Supplementary Table 2). A total of 22.88 × 10^3^ unigenes were annotated using the Trinity assembly program (Supplementary Table 3).

**Figure 2:**
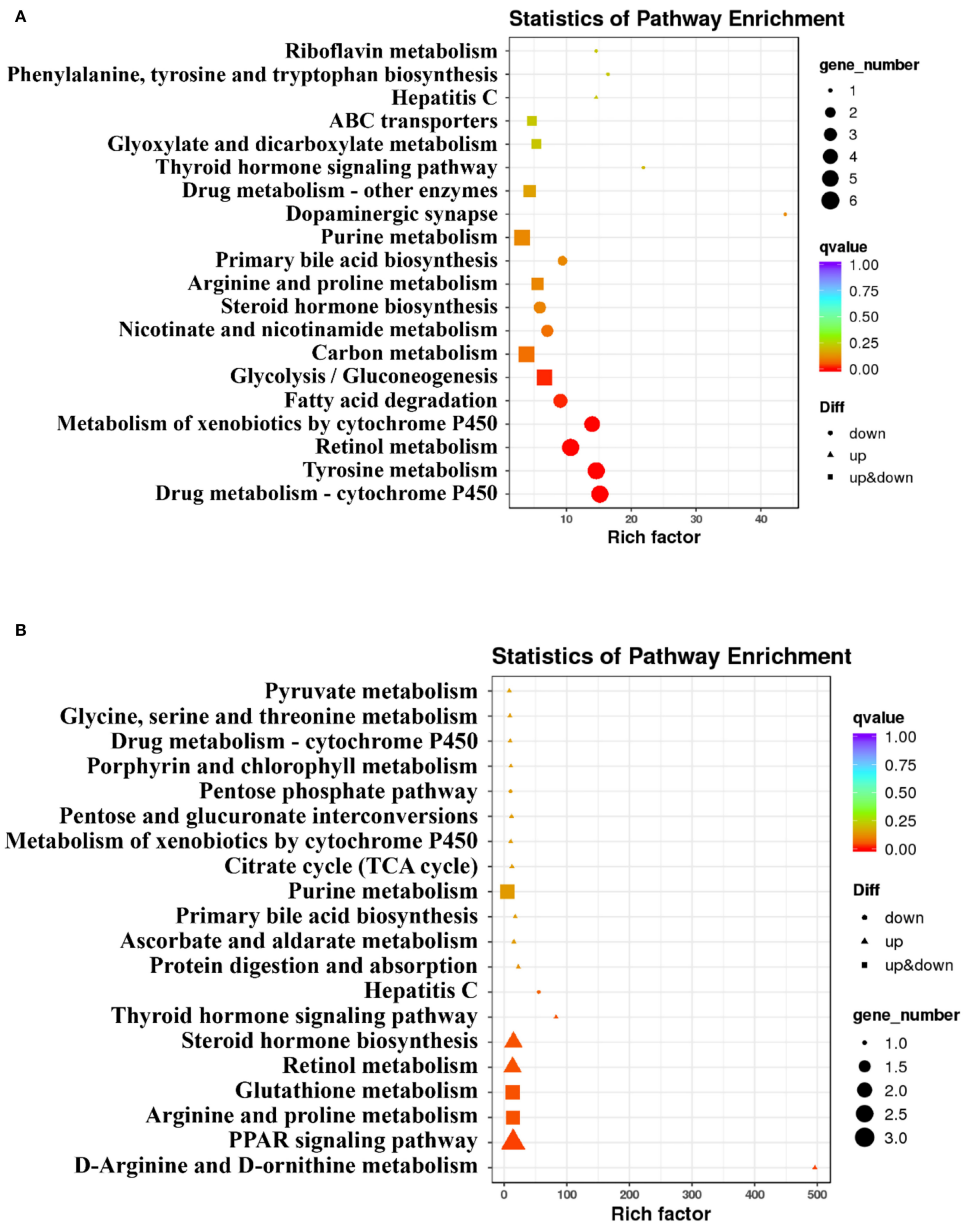
Analysis of Kyoto Encyclopedia of Genes and Genomes (KEGG) pathway on hepatic differentially expressed genes (DEGs) in swamp eel fed HC diet. **(A)** the HC group relative to the (normal carbohydrate) NC group, **(B)** the HC diet supplemented with 2.5% SJ (HC-S) group relative to the HC group.

There were 181 DEGs between the HC group and NC group, and 38 DEGs between the HC-S group and the HC group with the screening criteria that FC ≥1.5 and an FDR <0.05. To further functionally characterize the DEG, pathway analysis was conducted using the KEGG database. Through the KEGG pathway analysis of DEG, pathways in the top 20 significant pathways between the HC group and NC group, and the HC-S group and HC group are listed in Figures 2A,B, respectively. The changes of DEG in the top 20 significant pathways are as follows: in the aspect of carbohydrate metabolism, all the DEGs including the *acss1* and *adh* in glycolysis/gluconeogenesis were downregulated or upregulated in the HC group compared with the NC group (Figure 2). Moreover, regarding BA metabolism, all the DEGs including the *akr1d1* and *cyp7a1* in primary bile acid biosynthesis were downregulated in the HC group compared with the NC group, whereas all the DEGs including the *akr1d1* and *cyp7a1* in that pathway were upregulated in the HC-S group compared with the HC group; SJ supplemental diet upregulated the expression of *ugt*, all the DEGs in the pathway of drug metabolism-cytochrome P450 (Figure 2). In addition, the SJ supplemental diet downregulated the expression of *ifit1*, all the DEGs in the pathway of hepatitis C, while the HC diet upregulated that DEG (Figure 2).

**Figure 3:**
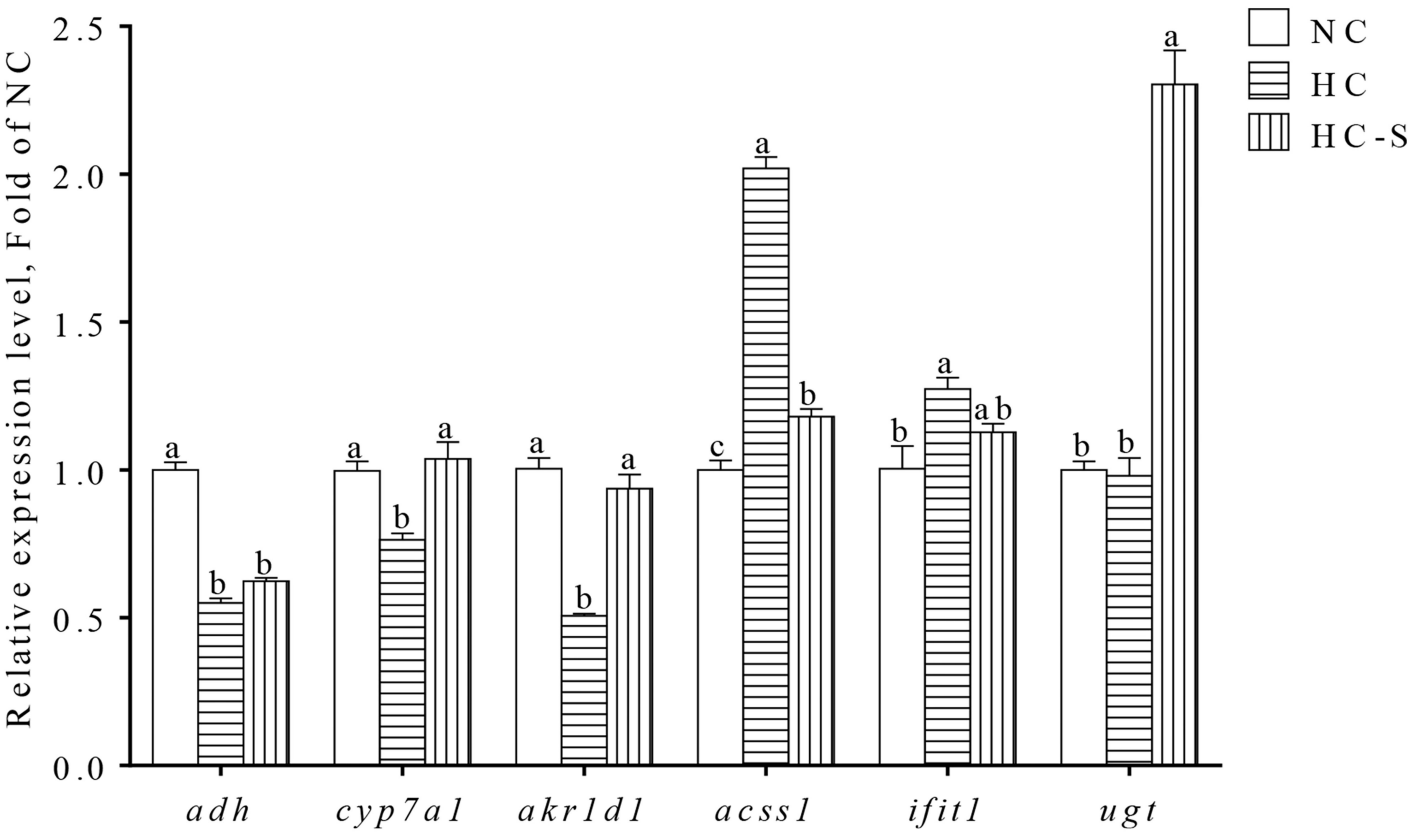
Quantitative real-time PCR (qPCR) analysis of hepatic DEGs in swamp eel fed HC diet. Values are presented as means ± SEM, *n* = 16 fish/diet. Columns with different superscripts indicated values with significant difference, *p* < 0.05 was significantly different.

Results of gene expression validation from qPCR are shown in Figure 3. Significantly lower expressions of *adh, cyp7a1*, and *akr1d1* were found in the HC group compared with the NC group, whereas the expression of *acss1* and *ifit1* showed the opposite change (*p* < 0.05). However, the changed expression of *adh, cyp7a1, akr1d1, acss1*, and *ifit1* in the HC group was ameliorated HC-S group. In addition, the expression of *ugt* was significantly upregulated in the HC-S group compared with the NC and HC groups (*p* < 0.05). The expression levels of DEGs (*adh, cyp7a1, akr1d1, acss1, ifit1*, and *ugt*) in qPCR showed a highly consistent trend from the results of the transcriptomic assay.

### Serum Profile of BA in Swamp eel Fed NC, HC, and HC-S Diet

**Figure 4:**
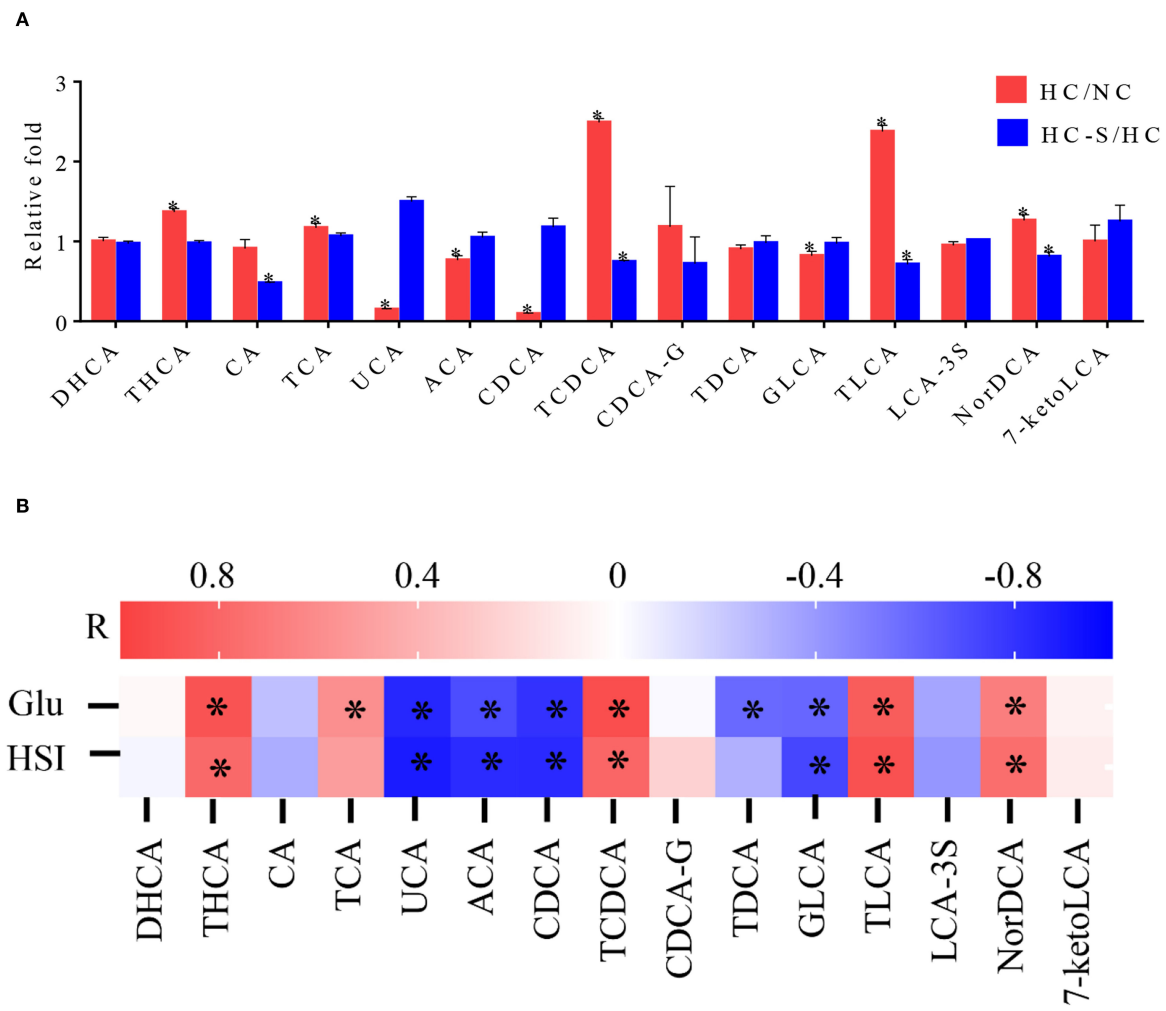
Influence of dietary SJ on the serum profile of bile acid (BA) in swamp eel fed HC diet. **(A)** Fold changes of 15 BA in the HC group relative to the mean values of the NC group and the HC-S group relative to the mean values of the HC group, **(B)** Heatmaps of Spearman's correlation coefficients of 15 BA with representative metabolic markers. In the bar plots **(A)**, *indicate the statistical significance (*p* < 0.05) between two groups. In the heatmaps **(B)**, *R* value indicates Spearman's correlation coefficient, and *indicates the statistical significance (*p* < 0.05) based on Spearman's correlation. Values are presented as means ± SEM, *n* = 16 fish/diet. THCA, taurohyocholic acid; CA, cholic acid; TCA, taurocholic acid; UCA, ursocholic acid; ACA, allocholic acid; CDCA, chenodeoxycholic acid; TCDCA, taurochenodeoxycholic acid; CDCA-G, chenodeoxycholic acid-glucuronide; TDCA, taurodeoxycholic acid; GLCA, glycolithocholic acid; TLCA, taurolithocholic acid; LCA-3S, lithocholic acid-3-sulfate; NorDCA, nordeoxycholicacid; 7-ketoLCA, 7-Ketolithocholic acid.

To evaluate the association between BA species and liver injury, we conducted targeted serum BA profiling analyses in the NC, HC, and HC-S groups by UPLC-MS analysis. Based on UHPLC/MS data, a total of 15 BA met the quality control criteria and were quantified. The alterations of the differential BA species were then compared between groups. The taurohyocholic acid (THCA), taurocholic acid (TCA), taurochenodeoxycholic acid (TCDCA), taurolithocholic acid (TLCA), and nordeoxycholicacid (NorDCA) were significantly increased, and ursocholic acid (UCA), allocholic acid (ACA), chenodeoxycholic acid (CDCA), and glycolithocholic acid (GLCA) were significantly decreased in the HC group compared with the NC group (*p* < 0.05) (Figure 4A). Oppositely, the TCDCA, TLCA, and NorDCA were significantly decreased (*p* < 0.05), and UCA, ACA, and CDCA were increased in the HC-S group compared with the HC group (*p* > 0.05) (Figure 4A). Moreover, pairwise Spearman's correlation analysis showed that serum THCA, TCDCA, TLCA, and NorDCA were positively correlated with HSI and serum GLU, whereas serum UCA, ACA, CDCA, and GLCA were inversely correlated with HSI and serum GLU (*p* < 0.05) (Figure 4B). These results indicated that serum BA of THCA, TCDCA, TLCA, NorDCA, UCA, ACA, CDCA, and GLCA were related to GLU metabolism and regulation.

## Discussion

Increasing dietary carbohydrates can reduce the protein percentage in the diet, thus lowering the rearing cost and decreasing the ammonia emission into the environment of aquatic animals (16). However, high dietary carbohydrate levels could decrease feed palatability and accelerate satiety, and increase the HSI and liver injury of fish (17). In this study, the feed coefficient (FC) of the HC group was lower than that of the NC group, whereas the HSI showed the opposite change. These findings implied an enhanced nutrients-sparing but the liver injury effect in this fish-fed HC diet. Importantly, the decreased HSI was observed in HC-S (HC supplemented with 2.5% SJ) group compared with the HC group. Similarly, our previous study showed that appropriate SJ supplementation in diet could sustain the liver health in black seabream, which could be linked predominantly to SJ fucoidan (14, 18).

People are still suffering from iodine deficiency disorder (IDD) in some regions around the world, so finding a cost-efficient and healthful iodine supplementation source is extremely necessary (19). It was reported that the flesh iodine content from fish could be improved by increasing dietary iodine (20–22). In addition, marine macroalgae, a potential source of natural mineral additives, meet the concept of sustainable, chemical-free, and organic farming (22). Our results showed that the HC-S diet significantly increased the muscle iodine contents in swamp eel, which is highly appealing to consumers.

Increased serum AST, ALT, and BA, and hepatic glycogen, BA, and collagen fiber are a symbol of liver injury in fish (23, 24). Since bile flow is reduced, BA accumulation in liver cells leads to oxidative stress, apoptosis, and subsequent damage to the liver parenchyma (25). In the present study, fish fed HC diet exhibited high serum GLU, AST, ALT, and BA, and hepatic glycogen, BA, and collagen fiber by biochemical and histological analysis, which were consistent with a previous study in *Megalobrama Amblycephala* (26) and *Micropterus salmoides* (4). Interestingly, our results suggest that SJ supplementation may ameliorate liver injury by reducing serum ALT, AST, and BA, and hepatic glycogen, BA, and collagen fiber in swamp eel. Consistently, several reports demonstrated that dietary SJ improved the structure and function of the liver, and decreased serum GLU and BA in mammals (7, 27). However, the effects and underlying mechanism of dietary SJ on liver injury caused by a high carbohydrate diet in fish remain elusive.

It has been reported that acetyl-coenzyme A synthetase (ACSS1), the mitochondrial form of the enzyme, converts acetate to acetyl-CoA in mitochondria, and acetate-activated intra-mitochondrially by ACSS1 can be readily oxidized to CO_2_ for energy derivation (28). ADH converts ethanol to the aldehyde in the liver of mammals (29). In this study, all DEGs in glycolysis/gluconeogenesis, members of the top 20 significantly enriched pathways, were *adh* and *acss1*, wherein in comparison to the NC group, the HC group upregulated the expression of *acss1*, and simultaneously downregulated the expression of *adh* by transcriptomic analysis. Accordingly, the HC diet could promote energy derivation by upregulating the expression of *acss1*, which was consistent with the lower FCR in the HC group. Low enzymatic activity of ADH induced hepatic fibrosis in mice (30). Moreover, activating ADH protects against acute alcohol-induced liver injury in mice (31). The results suggest that the HC diet induces hepatic fibrosis by decreasing the expression of *adh*.

In addition, IFIT1 is one of the proteins induced by viruses (32). SJ supplemental diet downregulated the expression of *ifit1*, all the DEGs in the pathway of hepatitis C (members of the top 20 significantly enriched pathway), while HC diet upregulated that DEG. This indicates the protecting role of SJ against viruses in the liver of swamp eel.

Bile acid plays important role in cholesterol, lipid, and even GLU homeostasis (33). However, a high concentration of BA is toxic, and excess BA accumulation induced hepatocyte injury and liver fibrosis in mammals (34) and fish (35). CYP7A1, the rate-limiting enzyme, is thought to be the major contributor to BA synthesis (36). However, excess BA accumulation inhibits the expression of *cyp7a1* (37). In this study, the expression of all the DEGs (*akr1d1* and *cyp7a1*, synthesizing BA) in the primary BA synthesis, members of the top 20 significantly enriched pathways, were downregulated in the HC group, whereas the expression of those DEGs was reversely upregulated in the HC-S group. This result indicates that the HC diet induces BA accumulation, and consequently inhibits the expression of *cyp7a1*. In addition, the accumulated BA was reduced in the HC-S group compared with the HC group, which could cause the upregulation of the expression of *cyp7a1* in this study.

Glucuronide conjugates represent up to 10% of the BA circulating pool in healthy people (38), and UGT catalyzes BA detoxification with the process of glucuronidation (39). In this study, SJ supplementation upregulated the expression of *ugt*, all the DEGs in the pathway of drug metabolism-cytochrome P450 (members of top 20 significantly enriched pathway). This increased upregulated expression of *ugt* may be attributable to the high BA binding capacity of polysaccharide in SJ (40).

Bile acid toxicity decreases as the number of its hydroxyl increases (41). The BA of UCA, ACA, and CDCA is multi-hydroxy primary bile acid with lower toxicity (42, 43). Strikingly, the changing trends of UCA, ACA, and CDCA were reversely correlated with that of serum GLU and HSI in this study. Moreover, the HC-S diet increased serum UCA, ACA, and CDCA, whereas the HC diet decreased those in this study. These results suggest that UCA, ACA, and CDCA play critical role in regulating GLU homeostasis and reducing liver injury in swamp eel.

In summary, a high-carbohydrate diet resulted in liver injury through increasing the HSI, hepatic glycogen, BA, and collagen fiber, whereas those were decreased by SJ supplemented in a HC diet. Thoroughly, SJ supplementation in the HC diet could improve liver injury, which may be attributed to the suppression of BA synthesis, and decrease in BA toxicity by promoting the process of glucuronidation and increasing the multi-hydroxy BA proportion with hepatic transcriptomic and BA-targeted metabolomic analysis.

## Data Availability Statement

The original contributions presented in the study are publicly available. This data can be found here: http://dx.doi.org/10.17632/4ntfwyr9x7.1.

## Ethics Statement

The animal study was reviewed and approved by all experiments were conducted according to the National Institutes of Health Guide for the Care and Use of Laboratory Animals (NIH Publications No. 8023) in China. This study was approved by the Animal Experiment Ethics Committee of Jiangxi Agricultural University.

## Author Contributions

CY contributed to the conceptualization, formal analysis, investigation, and writing—review & editing. LW involved in software. WC contributed to the investigation. WZ presented data curation. ZH involved in visualization. ZW provided resources. ZY provided data curation. MP involved in project administration. HH contributed to the supervision. QZ contributed to the conceptualization, project administration, and funding acquisition. All authors contributed to the article and approved the submitted version.

## Funding

This work was supported by the China Agriculture Research System of MOF and MARA (CARS-46) and the National Natural Science Foundation of China (No. 31360641).

## Conflict of Interest

The authors declare that the research was conducted in the absence of any commercial or financial relationships that could be construed as a potential conflict of interest.

## Publisher's Note

All claims expressed in this article are solely those of the authors and do not necessarily represent those of their affiliated organizations, or those of the publisher, the editors and the reviewers. Any product that may be evaluated in this article, or claim that may be made by its manufacturer, is not guaranteed or endorsed by the publisher.
