# Supplementary material for: Dietary Macroalgae Saccharina japonica Ameliorates Liver Injury Induced by a High-Carbohydrate Diet in Swamp Eel (Monopterus albus)
Source: Front Vet Sci. 2022 Jun 14;9:869369. doi: 10.3389/fvets.2022.869369 (PMC9237522; doi:10.3389/fvets.2022.869369)
Supplement: Supplementary file 1 [file Table_1.DOCX]

**Supplementary Tables**

**Table 1** Primers used for relative quantitative real-time PCR (qPCR) analysis.

| **Primer** | **Forward primer 5’-3’** | **Reverse primer 5’-3’** |
| --- | --- | --- |
| *β-actin* | CGTCTGTAGCAGAGAAGCTTAG | GCTCGTCTTACCTGTGTGAATA |
| *adh* | CCACCCTGAATTGTTGTGTAATG | TGAGTGAGGCGTTGAGAAAG |
| *cyp7a1* | CCATGGCTACACTACAGAGAAC | GTGGCCCATCATCGTATCTATC |
| *akr1d1* | CCCTAAAGGGACAACATCTGAG | TATGGCTTGACCCACTTCATAC |
| *acss1* | CTCTACTGCGTCTGGTCTTTATT | CTCAGCCTCTTCCTCGTTATTT |
| *ifit1* | GTCCTTACCTTGGGCACTATTC | AGAACGGAAGGGAAGAATCATAAA |
| *ugt* | GACAGCAGTTTGCAGAAAGAAG | GAGGTTGGAGAAGACACGTAAA |

**Table 2** Sequencing reads after filtering.

| **Sample** | **Clean Reads** **(Mb)** | **Clean Bases (Gb)** | **Q20 (%)** | **Q30 (%)** | **GC ratio (%)** |  |
| --- | --- | --- | --- | --- | --- | --- |
|  |  |  |  |  |  |  |
| NC | 21.53 | 6.41 | 98.24 | 94.89 | 48.07 |  |
| HC | 21.39 | 6.37 | 98.07 | 94.47 | 48.66 |  |
| HC-S | 20.88 | 6.22 | 98.17 | 94.72 | 49.01 |  |

**Table 3** Annotation result of each functional database.

| **Values** | **Nr** | **eggNOG** | **Swissprot** | **Pfam** | **KOG** | **KEGG** | **GO** | **COG** | **ALL** |
| --- | --- | --- | --- | --- | --- | --- | --- | --- | --- |
| Number, x10^3^n | 22.83 | 20.52 | 13.60 | 20.22 | 15.10 | 19.86 | 20.60 | 5.35 | 22.88 |
| Percentage, % | 90.20 | 81.00 | 53.70 | 79.90 | 59.70 | 78.40 | 81.40 | 21.10 | 90.40 |
